# Microbiological Profile of Sarecycline, a Novel Targeted Spectrum Tetracycline for the Treatment of Acne Vulgaris

**DOI:** 10.1128/AAC.01297-18

**Published:** 2018-12-21

**Authors:** George Zhanel, Ian Critchley, Lynn-Yao Lin, Nancy Alvandi

**Affiliations:** a Department of Medical Microbiology and Infectious Diseases, University of Manitoba, Winnipeg, Manitoba, Canada; b Allergan plc, Irvine, California, USA

**Keywords:** *Propionibacterium acnes*, acne vulgaris, antibiotics, doxycycline, microbiological profile, microbiome, minocycline, sarecycline, tetracycline

## Abstract

Sarecycline is the first narrow-spectrum tetracycline-class antibiotic being developed for acne treatment. In addition to exhibiting activity against important skin/soft tissue pathogens, sarecycline exhibits targeted antibacterial activity against clinical isolates of Cutibacterium acnes. In the current study, sarecycline was 16- to 32-fold less active than broad-spectrum tetracyclines—such as minocycline and doxycycline—against aerobic Gram-negative bacilli associated with the normal human intestinal microbiome. Also, reduced activity against Escherichia coli was observed *in vivo* in a murine septicemia model, with the 50% protective doses, or the doses required to achieve 50% survival, being >40 mg/kg of body weight and 5.72 mg/kg for sarecycline and doxycycline, respectively. Sarecycline was also 4- to 8-fold less active than doxycycline against representative anaerobic bacteria that also comprise the normal human intestinal microbiome. Additionally, C. acnes strains displayed a low propensity for the development of resistance to sarecycline, with spontaneous mutation frequencies being 10^−10^ at 4 to 8 times the MIC, similar to those for minocycline and vancomycin. When tested against Gram-positive pathogens with defined tetracycline resistance mechanisms, sarecycline was more active than tetracycline against *tet*(K) and *tet*(M) strains, with MICs ranging from 0.125 to 1.0 μl/ml and 8 μl/ml, respectively, compared with MICs of 16 to 64 μl/ml and 64 μl/ml for tetracycline, respectively. However, sarecycline activity against the *tet*(K) and *tet*(M) strains was decreased compared to that against the wild type, which demonstrated MICs ranging from 0.06 to 0.25 μl/ml, though the decrease in the activity of sarecycline against the *tet*(K) and *tet*(M) strains was not as pronounced as that of tetracycline. These findings support sarecycline as a narrow-spectrum tetracycline-class antibiotic that is effective for the treatment of acne, and further investigation into the potential reduced effects on the gut microbiome compared with those of other agents is warranted.

## Abstract (precis)

Sarecycline is the first narrow-spectrum tetracycline-class antibiotic being developed for acne treatment. In addition to exhibiting activity against important skin/soft tissue pathogens, sarecycline exhibits targeted antibacterial activity against clinical isolates of Cutibacterium acnes.

## INTRODUCTION

Tetracyclines have been widely used for the treatment of moderate to severe acne due to their ability to suppress the growth of Cutibacterium acnes—an anaerobic organism associated with acne lesions—and their ability to exert anti-inflammatory effects (1, 2). Although tetracycline was frequently used in the 1950s and 1960s, its use has been superseded by that of other tetracyclines, such as doxycycline and minocycline, due to their improved bioavailability, lipophilicity (improved uptake into the pilosebaceous unit), and longer half-lives, allowing less frequent dosing (3, 4). Doxycycline is currently preferred as the first-line oral tetracycline for the treatment of acne (5), as other systemic treatment approaches (with tetracyclines and nontetracyclines, such as minocycline, co-trimoxazole, quinolones, clindamycin, macrolides, and trimethoprim) are associated with significant side effects and a risk for resistance development (6).

The antimicrobial action of tetracyclines against C. acnes occurs via inhibition of protein synthesis (7). In addition, C. acnes also produces proteins/enzymes that play a role in inflammation (e.g., lipase), which would also be downregulated as a consequence of inhibition of protein synthesis and which may account for the anti-inflammatory properties observed with sarecycline and other tetracyclines (7, 8).

While several tetracycline agents are available for acne treatment, the superior efficacy of one agent over another has never been determined. Therefore, side effect profiles may serve as a primary consideration in the choice of therapy. Notably, in recent years, the role of the human microbiome in maintaining health (9–11) and the impact of broad-spectrum antibiotics on dysbiosis (12) have garnered increasing attention. As minocycline and doxycycline exhibit potent broad-spectrum antimicrobial activity beyond their targeted pathogens (13), their widespread use is associated with off-target antibacterial effects on the human microbiome (i.e., intestinal flora), which may manifest clinically as diarrhea, fungal overgrowth (in the intestine and vagina), and vaginal candidiasis, especially in patients undergoing acne treatment, which involves prolonged oral administration (the typical duration of oral doxycycline or minocycline therapy is ∼12 weeks) (14–16). A potential association with inflammatory bowel disease has also been attributed to the widespread use of doxycycline and minocycline (17). Furthermore, the increased and prolonged use of tetracyclines is associated with the development of antibiotic resistance (8), though the risk may be lower than that from erythromycin, which is also utilized in acne treatment (18).

**FIG 1:**
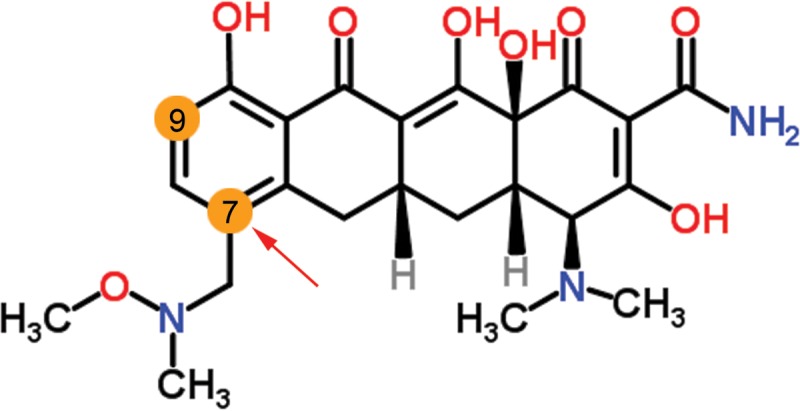
Structure of sarecycline. Sarecycline modification at C-7 (red arrow)–7-{[methoxy(methyl)amino]methyl}. The positions marked in orange at C-7 and C-9 have been modified to create tetracycline derivatives to potentially overcome tetracycline resistance mechanisms and to change bacterial ribosome binding. The figure is modified from that at http://www.chemspider.com/Chemical-Structure.28540486.html.

Sarecycline is a novel oral aminomethylcycline with a unique and stable modification at position C-7–7-{[methoxy(methyl)amino]methyl} (Fig. 1) and has recently completed evaluation in two phase 3 clinical trials, in which it was found to meet the 12-week primary efficacy endpoint for the treatment of moderate to severe acne (19, 20). The aim of the current study was to determine the spectrum of *in vitro* activity of sarecycline and comparator tetracyclines against clinical isolates of a broad panel of both aerobic and anaerobic bacteria, including C. acnes, and to assess its *in vivo* efficacy, mode of action, and potential for resistance development.

## RESULTS

### Activity against clinical isolates of C. acnes.

To assess the antibacterial activity of sarecycline and the comparators versus C. acnes, 55 clinical isolates of C. acnes were tested (the results are summarized in Table 1). The MIC values for sarecycline ranged from 0.5 to 16 µg/ml; the MIC_50_ was 0.5 µg/ml, and the MIC_90_ was 4 µg/ml. The comparator agents, which included tetracycline, doxycycline, and minocycline, exhibited similar antibacterial activity. A subset of the C. acnes isolates included organisms with high-level resistance to the macrolide erythromycin (MICs ≥ 128 µg/ml). All the tetracycline agents, including sarecycline, exhibited activity against the macrolide-resistant organisms (Table 2). Only one strain (strain 16099) exhibited an elevated sarecycline MIC of 16 µg/ml, and elevated MICs of the comparator tetracyclines were observed as well.

**TABLE 1:** Activity of sarecycline and comparator agents against 55 clinical isolates of C. acnes

Agent	MIC (µg/ml)		
	Range	50%	90%
Sarecycline	0.5 to 16	0.5	4
Tetracycline	0.5 to 32	1	2
Doxycycline	0.25 to 16	0.5	2
Minocycline	0.12 to 8	0.25	1
Clindamycin	≤0.06 to 64	≤0.06	4
Erythromycin	≤0.06 to >128	≤0.06	>128

**TABLE 2:** Activity of sarecycline and comparators against high-level erythromycin-resistant^*a*^ C. acnes clinical isolates

C. acnes strain	MIC (μg/ml)			
	Sarecycline	Tetracycline	Doxycycline	Minocycline
15758	4	8	4	2
16095	1	1	1	0.25
16099	16	32	16	8
16561	0.5	1	1	0.25
20660	4	4	2	1
20661	4	4	2	1
21368	4	4	2	1
21387	0.5	0.5	0.25	0.12
21388	0.5	1	0.5	0.25

a High-level erythromycin resistance was an erythromycin MIC of ≥128 µg/ml.

### Spectrum of activity.

Tetracyclines are known to have a broad spectrum of antibacterial activity when used for the treatment of acne, which includes activity against the normal microbiome (an unwanted off-target effect). Therefore, it was of interest to test the activity of sarecycline and the comparators against a broad collection of organisms encompassing the normal flora across the human body—including Gram-positive cocci, *Enterococcus* spp., *Enterobacteriaceae*, and Gram-positive and -negative anaerobes—to assess sarecycline’s spectrum of activity.

The activity of sarecycline and comparators against a broad collection of clinically important Gram-positive cocci is summarized in Table 3. Among the isolates of Staphylococcus aureus, sarecycline, like the other tetracyclines, maintained equivalent activity against both methicillin-susceptible and -resistant isolates, with an MIC_90_ value of 0.5 µg/ml. Sarecycline was also active against clinical isolates of Staphylococcus epidermidis, with MIC_90_ values of 2 µg/ml against both methicillin-susceptible and -resistant phenotypes. Sarecycline was more active than tetracycline and doxycycline against Staphylococcus haemolyticus, with an MIC_90_ value of 2 µg/ml, whereas the MIC_90_s of tetracycline and doxycycline were >32 and 16 µg/ml, respectively. Among the clinical isolates of Streptococcus pyogenes and Streptococcus agalactiae tested, sarecycline also exhibited activity equivalent to that of minocycline, with MIC_90_s of 8 µg/ml and 16 µg/ml, respectively.

**TABLE 3:** Activity of sarecycline and comparators against aerobic Gram-positive cocci

Organism (phenotype)	No. of isolates	Agent	MIC (µg/ml)		
			Range	50%	90%
S. aureus (methicillin susceptible)	32	Sarecycline	0.25 to 16	0.5	0.5
		Tetracycline	0.25 to >32	0.25	0.5
		Doxycycline	0.12 to 8	0.12	0.25
		Minocycline	0.06 to 8	0.12	0.12
S. aureus (methicillin resistant)	31	Sarecycline	0.25 to 4	0.25	0.5
		Tetracycline	0.25 to 2	0.25	0.5
		Doxycycline	0.12 to 2	0.12	0.25
		Minocycline	0.06 to 0.5	0.06	0.12
S. epidermidis (methicillin susceptible)	31	Sarecycline	0.12 to 2	0.25	2
		Tetracycline	0.12 to 2	0.25	2
		Doxycycline	0.06 to 1	0.12	1
		Minocycline	0.06 to 0.25	0.06	0.25
S. epidermidis (methicillin resistant)	33	Sarecycline	0.25 to 2	0.5	2
		Tetracycline	0.25 to >32	1	2
		Doxycycline	0.12 to 8	0.5	1
		Minocycline	0.06 to 0.5	0.12	0.25
S. haemolyticus	33	Sarecycline	0.12 to 2	0.12	2
		Tetracycline	0.12 to >32	1	>32
		Doxycycline	0.06 to 16	0.5	16
		Minocycline	≤0.03 to 0.5	0.06	0.5
S. pyogenes	32	Sarecycline	0.12 to 16	0.12	8
		Tetracycline	0.12 to 32	0.12	32
		Doxycycline	0.06 to 8	0.12	4
		Minocycline	0.03 to 8	0.06	8
S. agalactiae	31	Sarecycline	0.12 to 32	16	16
		Tetracycline	0.12 to >32	32	>32
		Doxycycline	0.06 to 16	8	16
		Minocycline	0.03 to 16	16	16
E. faecalis (vancomycin susceptible)	31	Sarecycline	0.5 to 32	32	32
		Tetracycline	0.25 to >64	32	64
		Doxycycline	0.12 to 16	8	8
		Minocycline	0.06 to 16	8	16
E. faecium (vancomycin resistant)	30	Sarecycline	0.12 to 32	2	32
		Tetracycline	0.12 to >64	2	>64
		Doxycycline	0.06 to 16	1	8
		Minocycline	≤0.03 to 16	0.25	16
E. faecium (vancomycin susceptible)	32	Sarecycline	0.12 to 32	0.5	32
		Tetracycline	0.12 to >64	1	>64
		Doxycycline	0.06 to 32	0.5	16
		Minocycline	≤0.03 to 16	0.12	16

The susceptibility results for sarecycline and the comparator agents against the *Enterococcus* spp. are also summarized in Table 3. Sarecycline had limited antibacterial activity against the vancomycin-susceptible isolates of Enterococcus faecalis, with an MIC_50_ value of 32 µg/ml, whereas the MIC_50_s of doxycycline and minocycline were 8 µg/ml. Although sarecycline was also less active against both vancomycin-susceptible and -resistant isolates of Enterococcus faecium than against the other species tested, it had activity that was either equivalent to or slightly less than that of the comparator tetracyclines.

**FIG 2:**
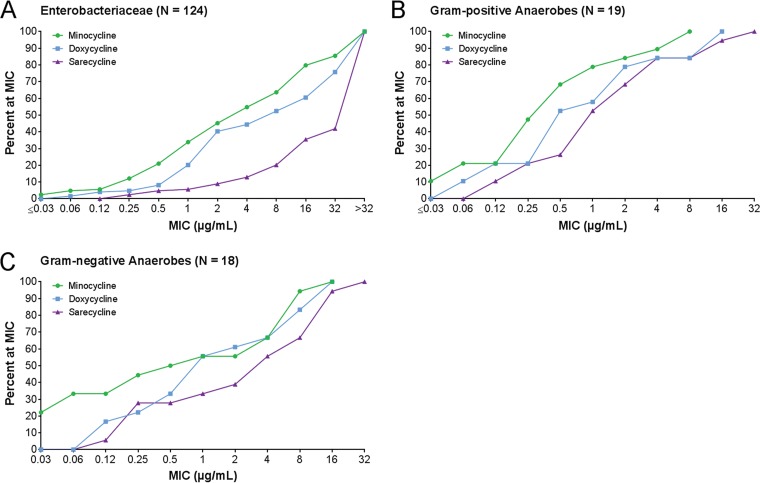
MIC distributions for sarecycline, doxycycline, and minocycline against *Enterobacteriaceae* (A), Gram-positive anaerobes (B), and Gram-negative anaerobes (C). (A) The isolates tested were E. cloacae (*n* = 30), E. coli (*n* = 33), K. pneumoniae (*n* = 31), and P. mirabilis (*n* = 30). (B) The isolates tested were B. bifidum (*n* = 1), B. brevi (*n* = 1), B. infantis (*n* = 1), B. longum (*n* = 1), C. perfringens (*n* = 2), C. difficile (*n* = 2), L. acidophilus (*n* = 1), L. casei (*n* = 1), L. plantarum (*n* = 1), P. anaerobius (*n* = 2), P. micros (*n* = 2), C. acnes (*n* = 2), S. constellatus (*n* = 1), and S. intermedius (*n* = 1). (C) The isolates tested were B. fragilis (*n* = 2), B. ovatus (*n* = 2), B. thetaiotaomicron (*n* = 2), B. vulgatus (*n* = 2), E. corrodens (*n* = 1), F. necrophorum (*n* = 1), F. nucleatum (*n* = 1), P. asaccharolytica (*n* = 2), P. melaninogenica (*n* = 2), *Prevotella* spp. (*n* = 2), and V. parvula (*n* = 1).

The activity of sarecycline and the comparator agents against common members of the *Enterobacteriaceae* is summarized in Table 4. Among the isolates of Enterobacter cloacae tested, sarecycline was the least active of the agents tested, with an MIC_50_ of 32 µg/ml, whereas the MIC_50_ of the other tetracyclines was 1 or 2 µg/ml. Similar results were observed for Escherichia coli, for which the MIC_50_ value for sarecycline was 16 µg/ml, whereas the MIC_50_ was 1 or 2 µg/ml for tetracycline, doxycycline, and minocycline. Sarecycline was largely inactive against Klebsiella pneumoniae clinical isolates, with MIC_50_ and MIC_90_ values that were >64 µg/ml. Sarecycline, in common with the other tetracyclines, was inactive against the isolates of Proteus mirabilis that were tested. Among the clinical isolates of *Salmonella* spp. tested, sarecycline had an MIC_50_ value of 16 µg/ml and was 8-fold less active than the other tetracyclines, for which the MIC_50_ values were 2 µg/ml. The comparative MIC distributions for all 124 clinical isolates of *Enterobacteriaceae* are shown as a Finlandogram in Fig. 2A. Sarecycline’s MIC distributions against the enteric Gram-negative bacteria were shifted to the right compared to those of minocycline and doxycycline, illustrating the 16- to 32-fold reduced potency of sarecycline based on comparisons of the MIC_50_ values.

**TABLE 4:** Activity of sarecycline and comparators against aerobic Gram-negative bacilli

Organism	No. of isolates	Agent	MIC (µg/ml)		
			Range	50%	90%
E. cloacae	30	Sarecycline	0.25 to >64	32	>64
		Tetracycline	0.5 to >64	2	>64
		Doxycycline	0.06 to >32	2	32
		Minocycline	≤0.03 to >32	1	16
E. coli	33	Sarecycline	2 to >64	16	>64
		Tetracycline	1 to >64	2	>64
		Doxycycline	0.5 to >32	2	32
		Minocycline	0.25 to >32	1	8
K. pneumoniae	31	Sarecycline	16 to >64	>64	>64
		Tetracycline	1 to >64	8	>64
		Doxycycline	1 to >32	8	>32
		Minocycline	1 to >32	4	>32
P. mirabilis	30	Sarecycline	>64	>64	>64
		Tetracycline	16 to >64	32	64
		Doxycycline	32 to >32	>32	>32
		Minocycline	8 to >32	16	>32
*Salmonella* spp.	35	Sarecycline	8 to >64	16	>64
		Tetracycline	1 to >64	2	>64
		Doxycycline	2 to >32	2	32
		Minocycline	1 to >32	2	8

**FIG 3:**
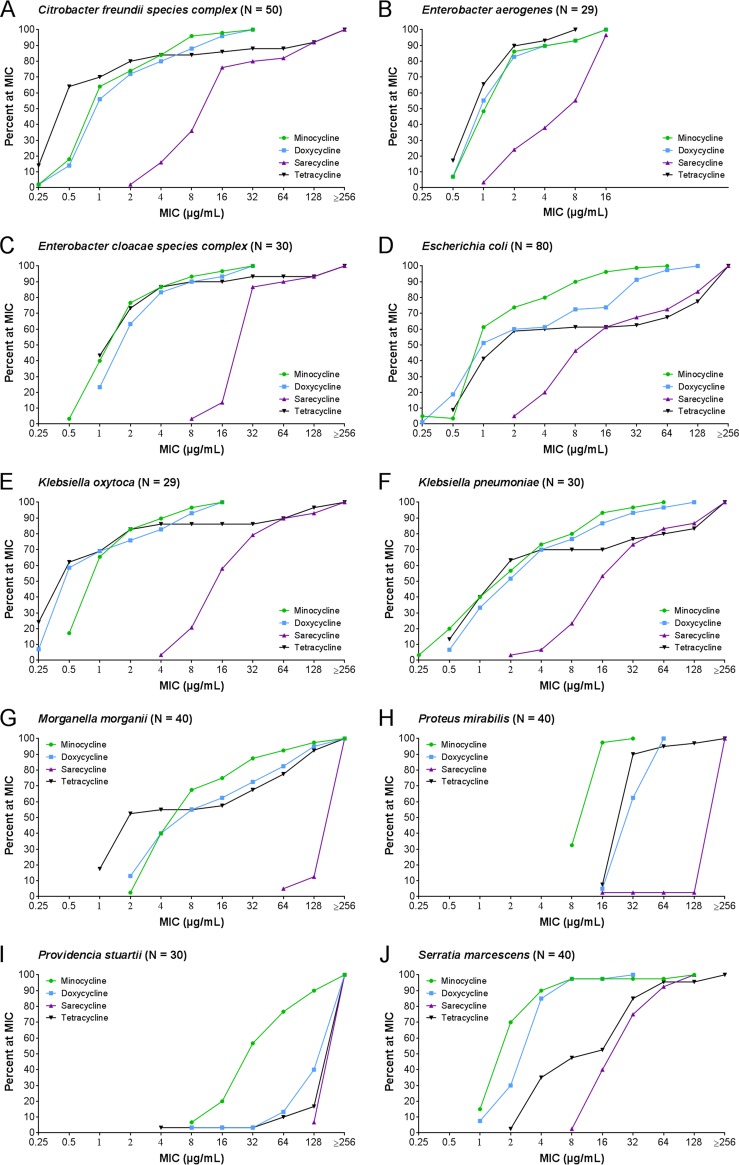
MIC distributions for sarecycline, doxycycline, and minocycline against contemporary clinical isolates of *Enterobacteriaceae* collected in 2015 and 2016 from patients 11 to 40 years old.

A separate study was also conducted to assess the activity of sarecycline and the comparators against 389 contemporary clinical isolates consisting of 10 members of the *Enterobacteriaceae* and the normal flora in the human gastrointestinal tract. The organisms were collected in 2015 and 2016 from patients at the common acne age of 11 to 40 years. The list of organisms and the activity of sarecycline are summarized in Table 5. Among the isolates of the Citrobacter freundii species complex, sarecycline was the least active agent tested, with an MIC_50_ and an MIC_90_ of 16 µg/ml and 128 µg/ml, respectively, whereas the MIC_50_ was 0.5 or 1 µg/ml for the other tetracyclines and the MIC_90_ was 8 and 16 µg/ml for minocycline and doxycycline, respectively. Tetracycline’s MIC_90_ value of 128 µg/ml was as low as that of sarecycline. Similar results were observed for E. coli, K. pneumoniae, and Klebsiella oxytoca, for which the MIC_50_ value for sarecycline was 16 µg/ml, whereas it ranged from 0.5 to 2 µg/ml for tetracycline, doxycycline, and minocycline. The MIC_90_ value of sarecycline against these species was 128 to 256 µg/ml, whereas it was 8 to 32 µg/ml for minocycline and doxycycline, but the lower activity of sarecycline was similar to that of tetracycline, which showed an MIC_90_ of 128 to 256 µg/ml. Against the isolates of Enterobacter aerogenes, sarecycline was also the least active agent, with an MIC_50_ and an MIC_90_ of 8 µg/ml and 16 µg/ml, respectively, whereas the MIC_50_ was 1 to 2 µg/ml and the MIC_90_ was 4 to 8 µg/ml for the other tetracyclines. Similar results were observed for the Enterobacter cloacae species complex, against which the MIC_50_ and MIC_90_ values of sarecycline were 32 µg/ml and 64 µg/ml, respectively, whereas the MIC_50_ and MIC_90_ of the other tetracyclines were 2 µg/ml and 8 µg/ml, respectively. For the isolates of Serratia marcescens, the MIC_50_ value of sarecycline was 32 µg/ml, whereas those of tetracycline, doxycycline, and minocycline were 2 to 16 µg/ml. The MIC_90_ value of sarecycline was 64 µg/ml, whereas the MIC_90_ values of minocycline and doxycycline were 4 and 8 µg/ml, respectively, but the lower activity of sarecycline was similar to that of tetracycline. Sarecycline showed no activity against the Morganella morganii and P. mirabilis clinical isolates tested, with MIC_50_ and MIC_90_ values of >256 µg/ml, whereas the MIC_50_ and MIC_90_ values of the other tetracyclines ranged from 2 to 128 µg/ml. Sarecycline, in common with the other tetracyclines, was largely inactive against the isolates of Providencia stuartii that were tested. The comparative MIC distributions for sarecycline and the comparators against 389 contemporary clinical isolates from 10 members of the *Enterobacteriaceae* and the normal flora found in the human intestinal track are shown as a Finlandogram in Fig. 3. Similar to the trend shown in Fig. 2A, sarecycline was generally 2- to >128-fold less potent than the other tetracyclines against all isolates tested when MIC_50_ values were compared. In addition, sarecycline showed 2- to 32-fold reduced potency compared to minocycline and doxycycline, based on comparisons of the MIC_90_ values against all isolates tested. At the MIC_90_, the lower activity of sarecycline was similar to that of tetracycline against the C. freundii species complex, E. coli, K. pneumoniae, K. oxytoca, P. stuartii, and S. marcescens, but it was >2-fold less active than tetracycline against the E. cloacae species complex, E. aerogenes, M. morganii, and P. mirabilis.

**TABLE 5:** Activity of sarecycline and comparators against aerobic Gram-negative bacilli collected in 2015 and 2016 from patients 11 to 40 years old

Organism (phenotype)	No. of isolates	Agent	MIC (µg/ml)		
			Range	50%	90%
C. freundii species complex	50	Sarecycline	2 to 256	16	128
		Tetracycline	0.25 to 256	0.5	128
		Doxycycline	0.25 to 32	1	16
		Minocycline	0.25 to 32	1	8
E. coli	80	Sarecycline	2 to >256	16	256
		Tetracycline	0.5 to >256	2	256
		Doxycycline	0.25 to 128	1	32
		Minocycline	0.25 to 64	1	8
K. pneumoniae	30	Sarecycline	2 to >256	16	256
		Tetracycline	0.5 to >256	2	256
		Doxycycline	0.5 to 128	2	32
		Minocycline	0.25 to 128	2	16
K. oxytoca	29	Sarecycline	4 to 256	16	128
		Tetracycline	0.25 to 256	0.5	128
		Doxycycline	0.25 to 16	0.5	8
		Minocycline	0.5 to 16	1	8
E. cloacae species complex	30	Sarecycline	8 to 256	32	64
		Tetracycline	1 to 256	2	8
		Doxycycline	1 to 32	2	8
		Minocycline	0.5 to 16	2	8
E. aerogenes	29	Sarecycline	1 to 128	8	16
		Tetracycline	0.5 to 16	1	4
		Doxycycline	0.5 to 16	1	8
		Minocycline	0.5 to 16	2	8
M. morganii	40	Sarecycline	64 to >256	>256	>256
		Tetracycline	1 to >256	2	128
		Doxycycline	2 to >256	8	128
		Minocycline	2 to 256	8	64
P. mirabilis	40	Sarecycline	16 to >256	>256	>256
		Tetracycline	16 to >256	32	32
		Doxycycline	16 to 64	32	64
		Minocycline	8 to 32	16	16
P. stuartii	30	Sarecycline	128 to >256	>256	>256
		Tetracycline	4 to >256	256	>256
		Doxycycline	8 to >256	256	256
		Minocycline	8 to 256	32	128
S. marcescens	40	Sarecycline	8 to 128	32	64
		Tetracycline	2 to 256	16	64
		Doxycycline	1 to 32	4	8
		Minocycline	1 to 128	2	4

Sarecycline was also tested against 19 representative Gram-positive anaerobic bacteria that included isolates of Bifidobacterium bifidum, Brevibacillus brevi, Bifidobacterium infantis, Bifidobacterium longum, Clostridium perfringens, Clostridium difficile, Lactobacillus acidophilus, Lactobacillus casei, Lactobacillus plantarum, Peptostreptococcus anaerobius, Peptostreptococcus micros, C. acnes, Streptococcus constellatus, and Streptococcus intermedius. The comparative MIC distributions for sarecycline and the comparators against all 19 Gram-positive anaerobes are shown as a Finlandogram in Fig. 2B. The MIC distributions for sarecycline are shifted to the right compared to those for minocycline and doxycycline, reflecting a 4- to 8-fold reduced potency against the representative Gram-positive anaerobes.

The MIC distributions for sarecycline and the comparators against 18 isolates of Gram-negative anaerobes were tested as well. These anaerobes included Bacteroides fragilis, Bacteroides ovatus, Bacteroides thetaiotaomicron, Bacteroides vulgatus, Eikenella corrodens, Fusobacterium necrophorum, Fusobacterium nucleatum, Peptostreptococcus assaccharolyticus, Prevotella melaninogenica, *Prevotella* spp., and Veillonela parvula. The comparative MIC distribution for sarecycline and the comparators against all 18 Gram-negative anaerobes are shown as a Finlandogram in Fig. 2C. Sarecycline was the least active tetracycline against the representative Gram-negative anaerobes.

### Effect on macromolecular biosynthesis.

**FIG 4:**
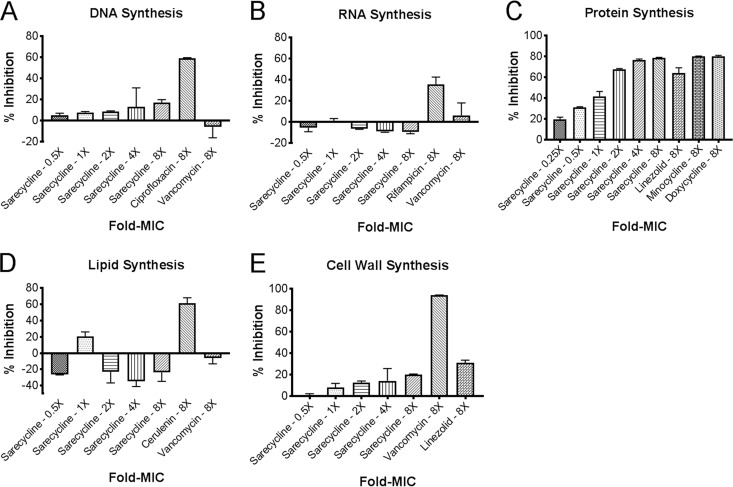
Effect of sarecycline on macromolecular biosynthesis in S. aureus ATCC 29213. DNA, RNA, protein, cell wall, and lipid synthesis was determined by measurement of the incorporation of [^3^H]thymidine, [^3^H]uridine, [^3^H]leucine, [^3^H]*N*-acetylglucosamine, and [^3^H]glycerol, respectively. Control agents included ciprofloxacin (a DNA synthesis inhibitor), linezolid (a protein synthesis inhibitor), cerulenin (a lipid synthesis inhibitor), vancomycin (a cell wall biosynthesis inhibitor), and rifampin (a RNA synthesis inhibitor). Data represent the median with 95% confidence intervals (*n* = 3).

Protein synthesis is a vital precursor to macromolecular biosynthesis, which is an essential component of microbial growth and homeostasis. Tetracyclines elicit their antimicrobial activity by targeting protein synthesis. To assess sarecycline’s mechanism of action in comparison to that of the other tetracyclines, the inhibitory effects of each molecule on major biosynthetic endpoints, including DNA, RNA, protein, lipid, and cell wall synthesis, were measured. The effects of sarecycline on macromolecular biosynthesis in S. aureus ATCC 29213 are summarized in Fig. 4. Sarecycline inhibited protein synthesis in a concentration-dependent manner at concentrations ranging from 0.25- to 8-fold the MIC, reaching maximum inhibition of 80% at 8-fold the MIC. Minocycline and doxycycline also demonstrated approximately 80% inhibition at 8-fold the MIC. DNA synthesis was inhibited by ≤10% at 4-fold the MIC of sarecycline, while 8-fold the MIC resulted in 20% inhibition. In contrast, ciprofloxacin (the positive control) resulted in 60% inhibition of DNA synthesis. Sarecycline had little or no effect on lipid biosynthesis, whereas cerulenin (the positive control) resulted in 70% inhibition at 8-fold the MIC. Sarecycline’s effect on cell wall biosynthesis was also limited, with maximum inhibition of 22% being achieved at 8-fold the MIC, whereas vancomycin (the positive control) resulted in 90% inhibition at 8-fold the MIC. No inhibition of RNA synthesis was observed with sarecycline at 8-fold the MIC, whereas rifampin (the positive control) resulted in 40% inhibition.

### *In vivo* efficacy.

To assess the *in vivo* efficacies of sarecycline, doxycycline, and minocycline against S. aureus RN450-1 and E. coli PBS1478, a murine systemic (intraperitoneal) infection model was utilized (the results are summarized in Table 6). Sarecycline, doxycycline, and minocycline were effective in treating the systemic infection caused by S. aureus, with 50% protective dose (PD_50_) values of 0.25, 0.3, and 0.03 mg/kg of body weight, respectively. In contrast, sarecycline did not demonstrate *in vivo* efficacy against the systemic infection caused by E. coli PBS1478, even at the highest dose of 40 mg/kg tested, whereas both minocycline and doxycycline exhibited protective effects at doses that were ≤7 mg/kg.

**TABLE 6:** Efficacy of sarecycline and comparators against S. aureus and E. coli in a murine systemic model of infection at 48 h postinfection

Antibacterial agent	S. aureus RN450-1		E. coli PBS1478	
	MIC (μg/ml)	PD_50_ (mg/kg)	MIC (μg/ml)	PD_50_ (mg/kg)
Sarecycline	0.06	0.25	4	40
Doxycycline	0.06	0.3	0.5	5.72
Minocycline	0.06	0.03	1	6.95

To assess the comparative efficacies of sarecycline and doxycycline against S. aureus RN450-1, a murine neutropenic thigh infection model was utilized to represent a tissue-based infection (the results are summarized in Table 7). Sarecycline achieved a 2-log_10_ reduction in the bacterial burden in the thigh at a dose comparable to that of doxycycline, with 50% effective dose (ED_50_) values of 8.23 and 8.32 mg/kg, respectively.

**TABLE 7:** Efficacy of sarecycline and doxycycline against S. aureus in a murine neutropenic thigh infection model

Agent	MIC (μg/ml)	PD_50_ (mg/kg)
Sarecycline	0.06	8.23
Doxycycline	0.06	8.31

### Spontaneous mutation frequency and antimicrobial resistance.

Single-step resistance development studies were performed, using three clinical isolates and one American Type Culture Collection (ATCC) isolate of C. acnes and a total of 4 clinical isolates of S. epidermidis (methicillin susceptible) and S. aureus (methicillin susceptible), to assess sarecycline’s propensity for resistance development. Sarecycline had low spontaneous mutation frequencies ranging from 10^−9^ to 10^−11^ for C. acnes at 4- and 8-fold the MIC, similar to those for the comparator agents minocycline and vancomycin (Table 8). Sarecycline also showed low spontaneous mutation frequencies ranging from 10^−9^ for S. aureus and 10^−8^ for S. epidermidis at 4- and 8-fold the MIC, similar to those for vancomycin (Table 9).

**TABLE 8:** Spontaneous mutational frequencies of C. acnes with sarecycline, vancomycin, and minocycline

C. acnes strain	Agent	Concn (µg/ml)	MIC multiple	Spontaneous mutation frequency
1286	Sarecycline	8	8	<7.5 × 10^−11^
		4	4	<7.5 × 10^−11^
	Vancomycin	4	8	<7.5 × 10^−11^
		2	4	<7.5 × 10^−11^
	Minocycline	2	8	<7.5 × 10^−11^
		1	4	<7.5 × 10^−11^
1713	Sarecycline	4	8	<1.35 × 10^−10^
		2	4	<1.35 × 10^−10^
	Vancomycin	4	8	<1.35 × 10^−10^
		2	4	<1.35 × 10^−10^
	Minocycline	2	8	<1.35 × 10^−10^
		1	4	<1.35 × 10^−10^
5004	Sarecycline	4	8	7.87 × 10^−10^
		2	4	7.87 × 10^−10^
	Vancomycin	4	8	<7.87 × 10^−10^
		2	4	<7.87 × 10^−10^
	Minocycline	2	8	<7.87 × 10^−10^
		1	4	<7.87 × 10^−10^
5030	Sarecycline	4	8	9.35 × 10^−10^
		2	4	1.4 × 10^−9^
	Vancomycin	4	8	<9.35 × 10^−10^
		2	4	<9.35 × 10^−10^
	Minocycline	2	8	<9.35 × 10^−10^
		1	4	<9.35 × 10^−10^

**TABLE 9:** Spontaneous mutational frequencies of S. aureus and S. epidermidis isolates with sarecycline and vancomycin

Test organism	Agent	Concn (µg/ml)	MIC multiple	Mean inoculum size (no. of CFU)	Spontaneous mutation frequency
S. aureus 0100	Sarecycline	4	8	1.74 × 10^8^	<5.75 × 10^−9^
		2	4	1.74 × 10^8^	8.62 × 10^−9^
	Vancomycin	8	8	1.74 × 10^8^	<5.75 × 10^−9^
		4	4	1.74 × 10^8^	<5.75 × 10^−9^
S. aureus 3869	Sarecycline	4	8	4.33 × 10^8^	2.31 × 10^−9^
		2	4	4.33 × 10^8^	2.31 × 10^−9^
	Vancomycin	8	8	4.33 × 10^8^	2.31 × 10^−9^
		4	4	4.33 × 10^8^	2.31 × 10^−9^
S. epidermidis 3703	Sarecycline	8	8	2.17 × 10^8^	<4.61 × 10^−8^
		4	4	2.17 × 10^8^	<4.61 × 10^−8^
	Vancomycin	8	8	2.17 × 10^8^	<4.61 × 10^−8^
		4	4	2.17 × 10^8^	4.61 × 10^−8^
S. epidermidis 3759	Sarecycline	2	8	3.27 × 10^8^	<3.06 × 10^−8^
		1	4	3.27 × 10^8^	<3.06 × 10^−8^
	Vancomycin	8	8	3.27 × 10^8^	<3.06 × 10^−8^
		4	4	3.27 × 10^8^	<3.06 × 10^−8^

Active efflux and ribosomal protection are two common mechanisms of tetracycline resistance that have been identified (21). Acquisition of the gene *tet*(K), *tet*(L), or *tet*(38) confers the ability for active efflux, whereas the acquisition of the gene *tet*(M), *tet*(O), *tet*(S), or *tet*(W) confers ribosomal protection (8, 22). The most common genes that confer tetracycline resistance among S. aureus strains are *tet*(M) and *tet*(K) in combination (23–26). In order to assess susceptibility to sarecycline in the presence of known tetracycline resistance mechanisms, a defined collection of tetracycline-resistant S. aureus strains in which resistance is conferred by *tet*(K), *tet*(M), and *tet*(38) was tested against comparator agents (the results are summarized in Table 10). Strains of S. aureus were utilized, as strains of C. acnes with tetracycline resistance mechanisms were not available at the time that the study was conducted. Sarecycline was more active than tetracycline against strains of the *tet*(K) genotype, with the MICs of sarecycline ranging from 0.12 to 0.5 µg/ml and those of tetracycline ranging from 16 to 65 µg/ml. However, in common with the other tetracyclines, sarecycline displayed elevated MICs against strains containing a combination of both *tet*(M) and *tet*(38).

**TABLE 10:** Activity of sarecycline and comparators against tetracycline-resistant S. aureus clinical isolates

S. aureus strain genotype	No. of isolates	MIC (μg/ml)			
		Sarecycline	Tetracycline	Doxycycline	Minocycline
Wild type	20	0.06–0.25	0.06–0.25	0.06–0.25	0.125–0.5
*tet*(K)	4	0.125–1	16–64	1–4	0.25–1
*tet*(M)	2	8	64	16	4
*tet*(38)	2	4	2–4	1–2	0.5
*tet*(M), *tet*(38)	2	16–32	64	8	8–16

## DISCUSSION

Tetracyclines such as doxycycline and minocycline have been widely used for acne treatment due to their improved bioavailability and lipophilicity over those of tetracycline, providing better tissue penetration, including uptake into the pilosebaceous unit, than tetracycline (27). Doxycycline and minocycline also exhibit more potent broad-spectrum antibacterial activity than the other tetracycline derivatives, along with better absorption from the gastrointestinal tract (4, 28, 29). Due to their potent broad-spectrum antibacterial activity, both doxycycline and minocycline are approved for multiple indications, in addition to acne treatment (13). However, recent advances in the characterization of the human intestinal microbiome have shed greater light on the impacts of the widespread use of oral broad-spectrum antibiotics, including the tetracyclines (30–32). Treatment with tetracyclines may predispose acne patients to the overgrowth of tetracycline-resistant (and, potentially, multidrug-resistant) organisms, including the overgrowth of Candida albicans, ultimately resulting in intestinal and vaginal dysbiosis (33–35). There are also reports that the widespread use of minocycline and doxycycline presents an increased risk for inflammatory bowel disease (17). Although sarecycline retains antibacterial activity against C. acnes and important skin pathogens, such as staphylococci, the results of the present study demonstrated reduced activity—compared to that of doxycycline and minocycline—against aerobic enteric Gram-negative bacteria, as well as representative anaerobes that comprise the normal intestinal flora.

Sarecyline’s unique narrow spectrum of antibacterial activity may result in reduced dysbiosis of the intestinal flora (and, potentially, vaginal flora), manifesting as reduced overgrowth of resistant bacteria and C. albicans yeast infections as well as reduced gastrointestinal adverse effects, such as diarrhea. Two pivotal identically designed, placebo-controlled, phase 3 trials including approximately 2,000 patients evaluated the efficacy and safety of once-daily sarecycline treatment at 1.5 mg/kg for 12 weeks in patients aged 9 to 45 years with moderate to severe facial acne vulgaris (19, 20). The rates of adverse events associated with the gastrointestinal tract, such as nausea, vomiting, and abdominal pain, were generally low, while the rate of diarrhea in patients treated with sarecycline was comparable to that in patients treated with placebo (19, 20). Additionally, vulvovaginal candidiasis and vulvovaginal mycotic infection were rare (19, 20). In contrast, other tetracycline-class antibiotics may be associated with gastrointestinal tract side effects, phototoxicity (typically seen with doxycycline), candidiasis, or vestibular side effects (observed with minocycline) (5), whereas sarecycline produced such side effects at low rates (19, 20). Furthermore, the administration of doxycycline, minocycline, and tetracycline has been associated with disruption of the gut microbiome (36–38). Additional studies of sarecycline testing its putative reduced effects on the intestinal microbiome by comparing its effects with those of doxycycline and minocycline using *in vitro* intestinal/gut models (39) or studies in acne patients or healthy volunteers would be beneficial for a more comprehensive assessment of the potential benefits of minimizing intestinal dysbiosis.

Acne remains one of the most prevalent skin conditions among adolescents (40). Although several treatment options are available (28), there are several challenges that remain, including limiting resistance among C. acnes isolates, simplifying treatment regimens, and developing new agents with more favorable safety profiles. Sarecycline is the first narrow-spectrum tetracycline-derived antibiotic that may reduce the potential for gastrointestinal dysbiosis, adverse effects, and concerns regarding resistance development during therapy.

## MATERIALS AND METHODS

### Bacterial isolates.

A total of 55 individual clinical isolates of C. acnes were obtained from a culture collection that was tested centrally at the R. M. Alden Research Laboratory (Culver City, CA). C. acnes isolates were collected from 2003 to 2010 from various clinical specimens, such as acne facial skin, diabetic foot infection skin, blood, and specimens from infection sites, such as abdominal abscesses and perirectal abscesses. The isolate collection also included a subset of isolates demonstrating high-level resistance to erythromycin (MICs ≥ 128 µg/ml). Among the other representative anaerobes tested in the profiling study were 19 Gram-positive isolates (including representative strains of Bifidobacterium bifidum, Bifidobacterium brevi, Bifidobacterium infantis, Bifidobacterium longum, Clostridium perfringens, Clostridium difficile, Lactobacillus acidophilus, Lactobacillus casei, Lactobacillus plantarum, Peptostreptococcus anaerobius, Peptostreptococcus micros, Streptococcus constellatus, and Streptococcus intermedius) and 18 Gram-negative anaerobic organisms (including representative strains of Bacteroides fragilis, Bacteroides ovatus, Bacteroides thetaiotaomicron, Bacteroides vulgatus, Eikenella corrodens, Fusobacterium necrophorum, Fusobacterium nucleatum. Porphyromonas asaccharolytica, Prevotella melaninogenica, *Prevotella* spp., and Veillonela parvula).

The aerobic Gram-positive clinical isolates tested included E. faecalis, Enterococcus faecium, Staphylococcus aureus, Staphylococcus epidermidis, Staphylococcus haemolyticus, Streptococcus pyogenes, Streptococcus agalactiae, and group C streptococci. The members of the *Enterobacteriaceae* evaluated were Enterobacter cloacae, Escherichia coli, Klebsiella pneumoniae, Proteus mirabilis, and *Salmonella* spp. All isolates were tested centrally at Micromyx, LLC (Kalamazoo, MI).

A separate set of contemporary *Enterobacteriaceae* clinical isolates included Citrobacter freundii species complex, Escherichia coli, Klebsiella pneumoniae, Klebsiella oxytoca, Enterobacter cloacae species complex, Enterobacter aerogenes, Morganella morganii, Proteus mirabilis, Providencia stuartii, and Serratia marcescens. All isolates were obtained from JMI Laboratories and were collected in 2015 and 2016 from patients 11 to 40 years old. All isolates were tested at Allergan plc (Irvine, CA).

### Antibiotics and *in vitro* susceptibility testing.

Sarecycline powder was provided by Allergan plc (Irvine, CA). Tetracycline, doxycycline, minocycline, clindamycin, and erythromycin were purchased from Sigma-Aldrich (St. Louis, MO) and were tested as comparator agents to benchmark the activity of sarecycline. All anaerobic bacteria were tested using the reference agar dilution method in accordance with CLSI guidelines (41). All aerobic bacteria were tested using the reference broth microdilution (BMD) method in accordance with CLSI guidelines (42).

### Mode of action.

Sarecycline and the comparator agents were evaluated for their effects on macromolecular biosynthesis (DNA, RNA, cell wall, protein, and lipid synthesis) in S. aureus ATCC 29213. For DNA, RNA, and protein synthesis, the effects of the test agents on the incorporation of [^3^H]thymidine (DNA), [^3^H]uridine, or [^3^H]leucine were studied. Sarecycline was tested at multiples of the MIC value in triplicate in 96-well microtiter plates. The S. aureus ATCC 29213 culture was used after the growth reached early exponential phase (optical density at 600 nm = 0.2 to 0.3) in either Mueller-Hinton broth (DNA synthesis) or M9 minimal medium (protein synthesis). Following a 5-min incubation of the bacterial culture containing sarecycline, either [^3^H]thymidine (DNA synthesis), [^3^H]uridine (RNA synthesis), or [^3^H]leucine (protein synthesis) was added at 0.5 to 1.0 μCi per reaction mixture. The reactions were allowed to proceed at room temperature for 15 to 30 min and then stopped by adding 12 μl of cold 5% trichloroacetic acid (DNA and RNA synthesis) or 5% trichloroacetic acid–2% Casamino Acids (protein synthesis). The reaction mixtures were incubated on ice for 30 min, and the samples were collected and counted using a Beckman LS 3801 liquid scintillation counter.

For cell wall biosynthesis, the test agents were evaluated for their effects on the incorporation of [^3^H]*N*-acetylglucosamine, while [^3^H]glycerol was used for lipid synthesis. In a process similar to that used for DNA synthesis, following a 5-min incubation of the bacterial culture containing sarecycline at room temperature, [^3^H]*N*-acetylglucosamine (0.5 μCi/reaction mixture) was added. The mixture was allowed to incubate for 30 min at room temperature, and samples were collected and counted using a Beckman LS 3801 liquid scintillation counter.

The control agents evaluated in the macromolecular synthesis assays included ciprofloxacin (a DNA synthesis inhibitor), linezolid (a protein synthesis inhibitor), cerulenin (a lipid synthesis inhibitor), vancomycin (a cell wall biosynthesis inhibitor), and rifampin (an RNA synthesis inhibitor).

### *In vivo* efficacy.

The murine systemic intraperitoneal infection model with E. coli strain 1478 and S. aureus strain RN450-1 was used to evaluate the *in vivo* efficacy of sarecycline and the comparator agents. E. coli strain 1478 was obtained from the Paratek Pharmaceuticals culture collection and was originally derived from Bristol-Myers Squibb strain SC8294. The S. aureus RN450-1 strain was produced by taking S. aureus RN450 from the Paratek Pharmaceuticals culture collection (original strain, NCTC8325) and passaging it *in vivo* to increase its virulence, and then the S. aureus RN450-1 strain was cultured from blood collected from mice infected with S. aureus RN450. Six-week-old specific-pathogen-free male CD-1 mice weighing 18 to 30 g (Charles River, Hartford, CT) were used for all experiments.

For the S. aureus RN450-1 infection, the bacterial culture grew overnight in Mueller-Hinton broth to approximately 1 × 10^9^ CFU/ml. Serial dilutions of the bacterial suspension were performed in phosphate-buffered saline (PBS; Fisher Scientific, Boston, MA) to obtain the infectious dose. Septicemia was induced by infecting mice intraperitoneally with 3.5 × 10^6^ to 7.4 × 10^8^ CFU of bacteria in PBS with a 5% bacteriological mucin (VWE Scientific, Pittsburg, PA) suspension. The inoculum represented approximately 100-fold the 50% lethal dose (LD_50_). At 1 h postinfection, the mice were treated with a single dose of sarecycline, doxycycline, and/or minocycline administered subcutaneously at doses ranging from 0.01 to 0.5 mg/kg in a vehicle of sterile water. All drug doses were adjusted to account for the percentage of the active moiety.

For the E. coli infection, the bacterial culture grew overnight in Mueller-Hinton broth to approximately 2 × 10^9^ CFU/ml. Serial dilutions of the bacterial suspension were performed in PBS to obtain the infectious dose. An inoculum of 6.5 × 10^5^ to 1.6 × 10^7^ CFU was inoculated intraperitoneally and represented approximately 100 times the 50% lethal dose (LD_50_). At 1 h postinfection, the animals were treated with sarecycline, doxycycline, or minocycline subcutaneously at doses ranging from 0.5 to 40 mg/kg in a vehicle of sterile water. All drugs doses were adjusted to account for the percentage of the active moiety. Sarecycline was tested at a high dose of 40 mg/kg to evaluate efficacy against an enteric Gram-negative organism, such as E. coli. For both organisms, the 50% protective dose (PD_50_) was assessed at 48 h postinfection. The PD_50_ was defined as the dose required to achieve 50% survival.

A murine neutropenic thigh wound infection model was also utilized. Female SD-1 mice were rendered neutropenic by injecting cyclophosphamide (Sigma-Aldrich, St. Louis, MO) at 150 and 100 mg/kg on days −4 and −1 before infection, respectively. Severe neutropenia (<100 neutrophils/mm^3^) developed by day 0, when the infection studies were initiated. Before infecting the mice, S. aureus RN450-1 from a frozen stock was cultured overnight. By following the same inoculum preparation procedures described above, 1 × 10^5^ CFU/mouse of S. aureus RN450-1 was injected intramuscularly into the left thigh. At 2 and 6 h postinfection, sarecycline or doxycycline therapy was administered intravenously to each mouse at a dose of 0.33, 1, 3, or 9 mg/kg in a vehicle of sterile water. At 24 h, the thighs were removed and S. aureus RN450-1 was cultured in Trypticase soy agar (TSA) II plates with 5% sheep blood (Northeast Labs, Waterville, ME) from thigh tissue homogenate. The bacterial burden was assessed to determine the 50% effective dose (ED_50_), defined as the dose required to achieve a 2-log_10_ reduction in the bacterial burden compared with that for the nontreated control. The genotype information for the S. aureus RN450-1 and E. coli 1478 strains tested in these studies was not available.

### Resistance development.

Single-step resistance development studies were conducted *in vitro*. C. acnes was grown on brucella agar for 48 h under anaerobic conditions and suspended in brucella broth. For C. acnes, 4 different isolates, including three clinical isolates and one American Type Culture Collection (ATCC) isolate, were evaluated by plating 10^9^ to 10^10^ organisms onto brucella agar medium containing 4- or 8-fold the MIC of sarecycline, minocycline, or vancomycin. Spontaneous mutation frequencies were determined by enumerating the viable colonies after 48 h of incubation at 35°C.

The development of spontaneous resistance to sarecycline and vancomycin in Gram-positive clinical isolates which were the members of the normal human flora was also studied.

Two clinical isolates of Staphylococcus epidermidis (methicillin susceptible) and 2 clinical isolates of Staphylococcus aureus (methicillin susceptible) were evaluated in a single-step resistance development study by plating 10^8^ organisms onto Trypticase soy agar (TSA) plates. For each isolate, 4- or 8-fold the MIC was tested for sarecycline and vancomycin.

A collection of clinical isolates of S. aureus strains with known tetracycline resistance mechanisms—*tet*(K), *tet*(M), and *tet*(38)—was tested to assess the sarecycline susceptibility of these organisms. The broth microdilution (BMD) method was performed starting with growth of the isolates in Mueller-Hinton broth (Northeast Labs) to the density of a 0.5 McFarland standard. Appropriate dilutions of sarecycline or the comparator compounds from primary stock solutions were made in cation-adjusted Mueller-Hinton broth (BBL) to concentrations ranging from 0.06 to 64 µg/ml and were used in the assays. The culture turbidity was checked after 18 to 24 h of incubation at 35°C. The susceptibility of the bacteria to sarecycline and the comparator agents was analyzed.
